# Inhibition of stearoyl-coenzyme A desaturase 1 ameliorates hepatic steatosis by inducing AMPK-mediated lipophagy

**DOI:** 10.18632/aging.103082

**Published:** 2020-04-23

**Authors:** Youping Zhou, Li Zhong, Shengjie Yu, Wei Shen, Can Cai, Huihong Yu

**Affiliations:** 1 Department of Gastroenterology, The First Affiliated Hospital of Chongqing Medical University, Chongqing, China; 2 Department of Gastroenterology and Hepatology, The Second Affiliated Hospital of Chongqing Medical University, Chongqing, China; 3 Department of Urology, The Second Affiliated Hospital of Chongqing Medical University, Chongqing, China

**Keywords:** SCD1, AMPK, lipophagy, NAFLD

## Abstract

SCD1 is a key enzyme controlling lipid metabolism and a link between its activity and NAFLD has been proposed. Lipophagy is a novel regulatory approach to lipid metabolism regulation, which is involved in the development of NAFLD. However, the possible functional connection between SCD1 and lipophagy in NAFLD remains unknown. To investigate the molecular mechanisms through which SCD1 regulates lipophagy in hepatic steatosis, the model of hepatic steatosis was established by inducing mouse primary hepatocytes with sodium palmitate and feeding C57BL/6 mice with HFD. Our results indicated that sodium palmitate-treated hepatocytes exhibited increased SCD1 expression, AMPK inactivation and defective lipophagy. Inhibition of SCD1 expression in hepatocytes resulted in enhanced AMPK activity and lipophagy, and reduced lipid deposition. Although SCD1 overexpression led to decreased AMPK activity and lipophagy, lipid deposition was increased in hepatocytes. SCD1 regulated lipophagy through AMPK to affect lipid metabolism in mouse primary hepatocytes. Additionally, compared to HFD-fed mice, CAY10566(an SCD1-specific inhibitor)-treated mice exhibited significantly decreased hepatic steatosis and hepatic lipid droplet accumulation, as well as enhanced AMPK activity and lipophagy. This study elucidated that SCD1 inhibition ameliorates hepatic steatosis by inducing AMPK-mediated lipophagy, suggesting that the SCD1-AMPK-lipophagy pathway is a potential therapeutic target for NAFLD.

## INTRODUCTION

Nonalcoholic fatty liver disease (NAFLD) is currently considered the most common cause of chronic liver disease worldwide with an estimated overall global prevalence of 25% [1]. The imbalance between the synthesis and lipolysis of TGs is a key pathogenic process in the occurrence and development of NAFLD [2]. However, the precise mechanisms remain poorly understood and there is currently no effective method for preventing abnormal lipid accumulation in hepatocytes. Therefore, a better knowledge of the prevention of aberrant lipid deposition in hepatocytes is important.

Stearoyl-coenzyme A desaturase 1 (SCD1), a key enzyme in lipid metabolism, involves the regulation of TG synthesis and fatty acid oxidation in hepatocytes [3, 4]. Previous studies have suggested that NAFLD individuals had increased SCD1 activity [5, 6] and deletion of SCD1 gene had decreased the liver lipid synthesis [7], which indicated that SCD1 had close relationship with NAFLD. Autophagy is an intracellular catabolic process that plays a critical role in maintaining cellular homeostasis and normal mammalian physiology [8]. Singh et al. provided the first report that lipid droplets can be degraded in hepatocytes by a specific selective autophagy program, which was termed lipophagy [9]. When hepatocytes are stimulated by lipids, lipophagy is enhanced as a protective mechanism, resulting in increased lipid mobilization [10]. However, lipophagy is inhibited in the high-fat environment of NAFLD, which leads to reduced lipid mobilization and exacerbation of hepatic steatosis, while excessive lipid deposition further augments lipophagy and forms a “vicious circle” [11, 12]. Therefore, finding a target to break this “vicious circle” is greatly significant for understanding the mechanism and finding way to prevent or treat NAFLD. AMP-activated protein kinase (AMPK) is not just a sensor of balancing the cellular energy status, but it also plays critical roles in regulating lipid metabolism: activated AMPK can inhibit lipogenesis, increase fatty acid oxidation, and affect cholesterol and total TG synthesis [12–14]. Moreover, AMPK is a major autophagy regulator, involving in autophagy initiation, efficient autophagosome maturation and lysosomal fusion [15–17]. Furthermore, the change in AMPK activity is regulated by SCD1 activity: SCD1 inhibition increases AMPK phosphorylation and leads to changes in the expression of genes involved in lipogenesis [18]. Our previous study demonstrated that inhibition of SCD1 led to autophagy-induced apoptosis via AMPK signaling in human hepatocellular carcinoma (HCC) cells [19].

So, in the present study, we conducted the model of hepatic steatosis to investigated whether SCD1 could control lipophagy via AMPK to regulate hepatic lipogenesis so as to explore the mechanism and the potential therapeutic target of NAFLD.

## RESULTS

### Expression of SCD1 and activation of AMPK and lipophagy in sodium palmitate-induced primary hepatocytes

**Figure 1:**
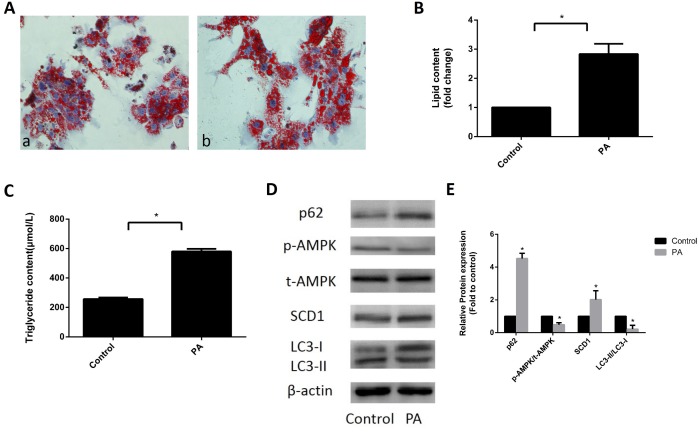
**Expression of SCD1 and activation of AMPK and lipophagy in sodium palmitate-treated primary hepatocytes.** (**A**) Primary hepatocytes were stained with Oil Red O and observed under an optical microscope. *a,* control group; *b,* PA group. (**B**) The intracellular lipid content in each group was quantified. (**C**) TG levels were measured with an enzymatic assay kit. (**D**, **E**) Protein levels were determined by Western blotting. The data are presented as the means±SDs. *p < 0.05 versus control.

Primary hepatocytes were treated with 324 μM sodium palmitate for 24 h. As shown in Figure 1A and 1B, the accumulation of lipid droplets was significantly increased in sodium palmitate-treated primary hepatocytes. In addition, the levels of intracellular TGs in sodium palmitate-treated primary hepatocytes were significantly higher than those in control hepatocytes (Figure 1C). These data indicated that 324 μM sodium palmitate could successfully induce hepatic steatosis. We further determined the expression patterns of SCD1, and AMPK and the function of lipophagy in hepatic steatosis. Western blot analysis revealed that SCD1 expression was increased in sodium palmitate-treated hepatocytes. However, total AMPK protein expression was not changed and phospho-AMPK (Thr172) levels were decreased in sodium palmitate-treated hepatocytes compared with those in control hepatocytes (Figure 1D, 1E). To investigate the means by which hepatic steatosis influenced lipophagy, we assessed the conversion of endogenous microtubule associated protein I light chain 3 (LC3-I) to LC3-II and the expression of p62, an autophagic substrate receptor, in sodium palmitate-treated hepatocytes. Sodium palmitate treatment resulted in an evident decrease in the conversion of LC3-I to LC3-II. Conversely, the expression of p62 was significantly elevated in sodium palmitate-treated hepatocytes (Figure 1D, 1E). Thus, these data suggested the upregulation of SCD1, decreased AMPK activation and defective lipophagy in hepatic steatosis.

### Effects of inhibited SCD1 expression on lipid deposition and activation of AMPK and lipophagy in primary hepatocytes

**Figure 2:**
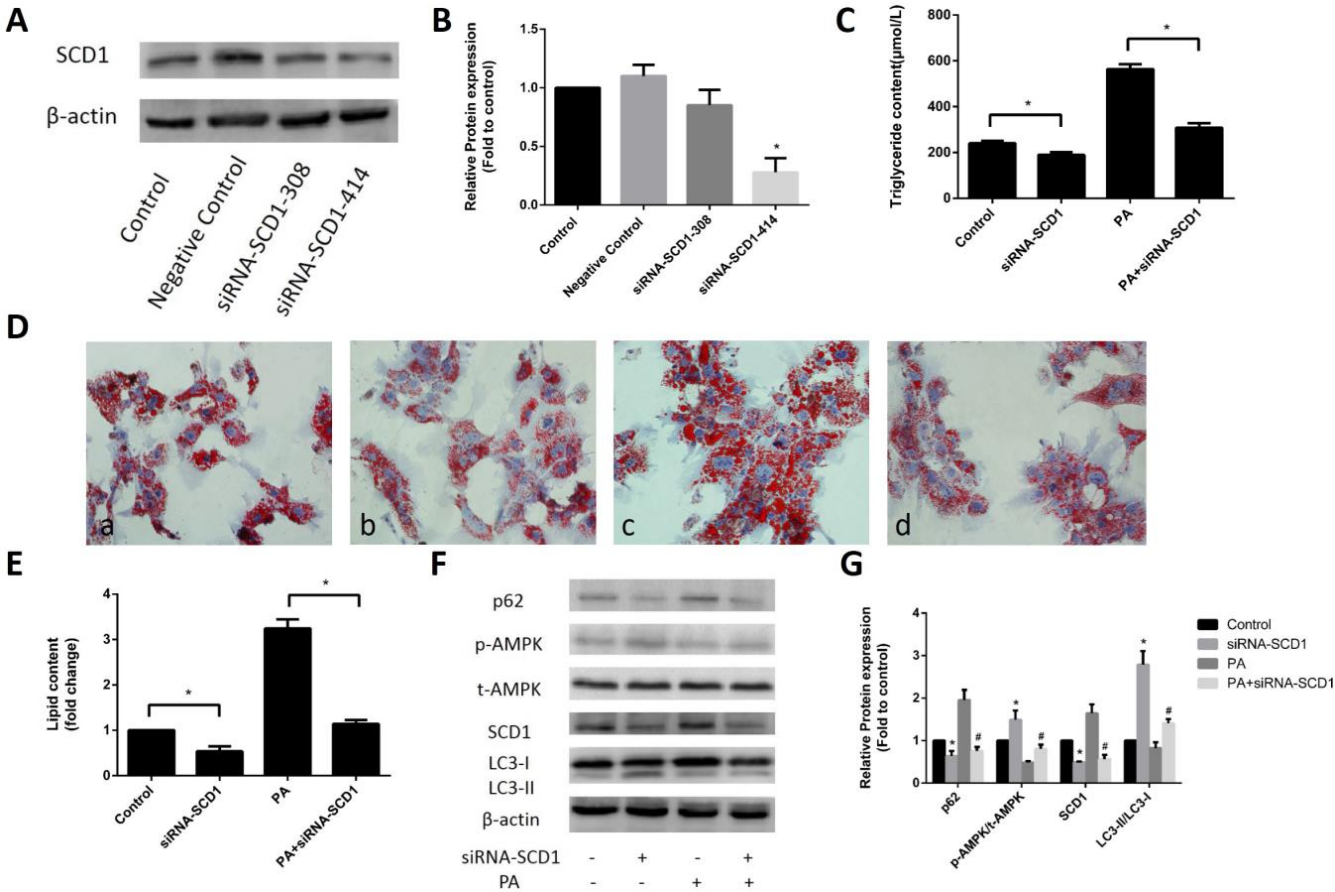
**Effects of inhibited SCD1 expression on lipid deposition and activation of AMPK and lipophagy in primary hepatocytes.** (**A**, **B**) Screening for the appropriate siRNA-SCD1 by Western blotting. (**C**) TG levels were measured after transfection with siRNA-SCD1. (**D**) Primary hepatocytes were stained with Oil Red O. *a,* control group; *b,* siRNA-SCD1 group; *c,* PA group; *d,* PA+siRNA-SCD1 group. (**E**) The intracellular lipid content in each group was quantified. (**F**, **G**) Protein levels were determined by Western blotting. The data are presented as the means±SDs. *p < 0.05 versus control, #*p < 0.05* versus the PA group.

To investigate whether SCD1 expression affects the sodium palmitate-induced reduction in AMPK phosphorylation and lipophagy, we first inhibited SCD1 expression in primary hepatocytes. As shown in Figure 2A, 2B, in primary hepatocytes transfected with siRNA-SCD1-308 or siRNA-SCD1-414, the latter siRNA significantly suppressed SCD1 activity and was selected for use in the following experiments. siRNA-SCD1 reduced the increase in intracellular TG levels (Figure 2C) and the accumulation of lipid droplets (Figure 2D, 2E) induced by sodium palmitate, indicating that inhibition of SCD1 activity can ameliorate hepatic steatosis in sodium palmitate-treated hepatocytes. We then examined AMPK protein expression and lipophagy. AMPK phosphorylation was significantly increased in hepatocytes treated with siRNA-SCD1, while total AMPK protein expression was not changed. siRNA-SCD1 enhanced the conversion of LC3-I to LC3-II, but decreased the expression of p62 in sodium palmitate-treated hepatocytes (Figure 2F, 2G).

### Effects of SCD1 overexpression on lipid deposition and activation of AMPK and lipophagy in primary hepatocytes

**Figure 3:**
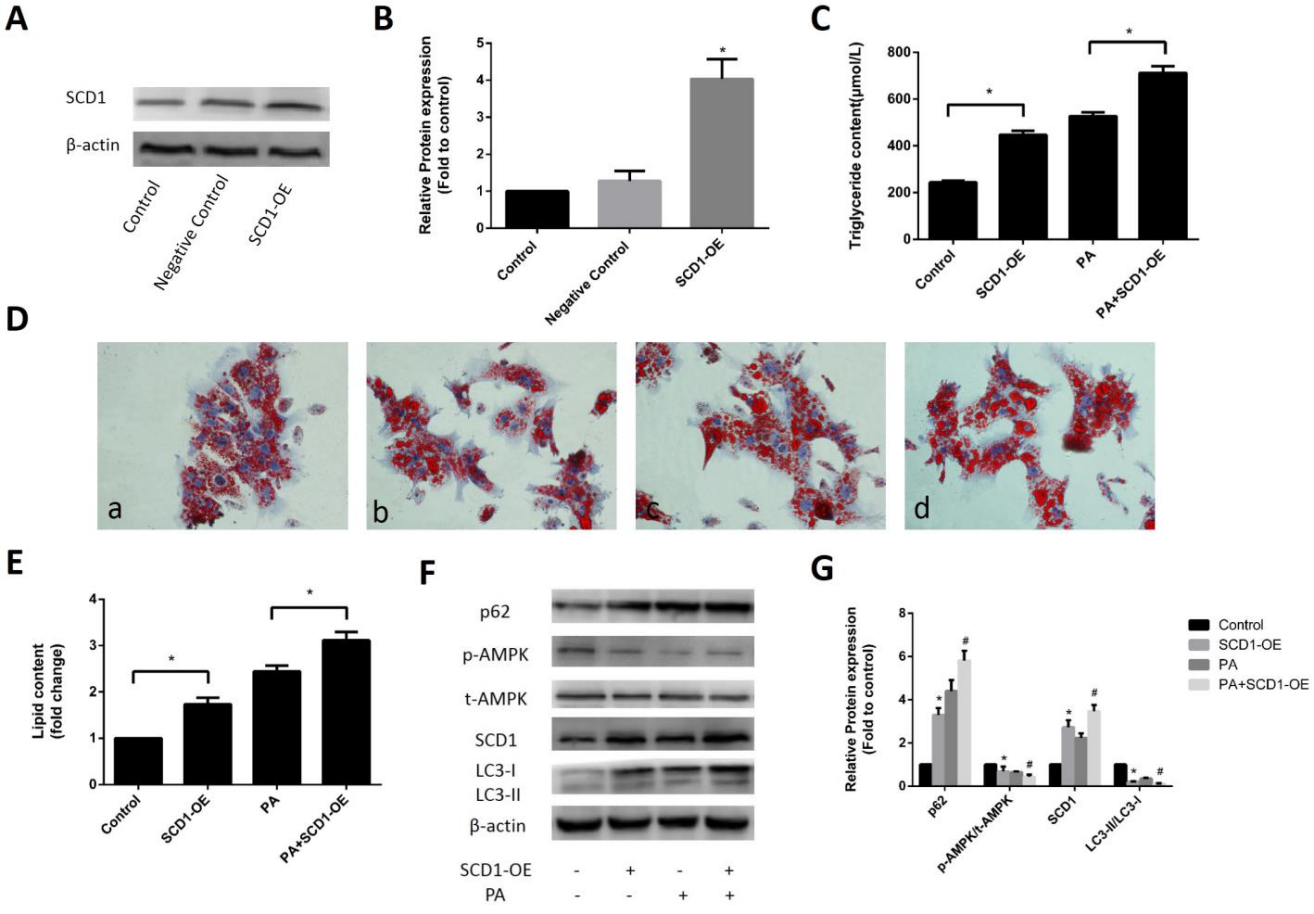
**Effects of SCD1 over-expression on lipid deposition and activation of AMPK and lipophagy in primary hepatocytes.** (**A**, **B**) The effect of SCD1-OE infection was verified by Western blotting. (**C**) TG levels were measured after infection with SCD1-OE. (**D**) Primary hepatocytes were stained with Oil Red O. *a,* control group; *b,* SCD1-OE group; *c,* PA group; *d,* PA+SCD1-OE group. (**E**) The intracellular lipid content in each group was quantified. (**F**, **G**) Protein levels were determined by Western blotting. The data are presented as the means±SDs. *p < 0.05 versus control, #*p < 0.05* versus the PA group.

To further evaluate the effect of SCD1 overexpression on sodium palmitate-treated hepatocytes, we infected primary hepatocytes with SCD1-OE, and induced the cells with sodium palmitate. As shown in Figure 3A, 3B, SCD1-OE infection could significantly increased the protein expression of SCD1. Regardless of whether hepatocytes were stimulated with sodium palmitate, the intracellular TG levels (Figure 3C) and lipid droplet accumulation were increased by SCD1-OE infection (Figure 3D, 3E). Western blotting showed that in contrast to the control group, hepatocytes infected with SCD1-OE exhibited significantly decreased AMPK phosphorylation, while total AMPK protein expression was not changed. The conversion of LC3-I to LC3-II in hepatocytes over expressing SCD1 was significantly decreased compared with that in hepatocytes treated with sodium palmitate alone. In addition, the expression of p62 in hepatocytes over expressing SCD1 was higher than that in hepatocytes treated with sodium palmitate alone (Figure 3F, 3G).

### Effects of cotreatment with siRNA-SCD1 and the AMPK inhibitor on lipid deposition and lipophagy in primary hepatocytes

**Figure 4:**
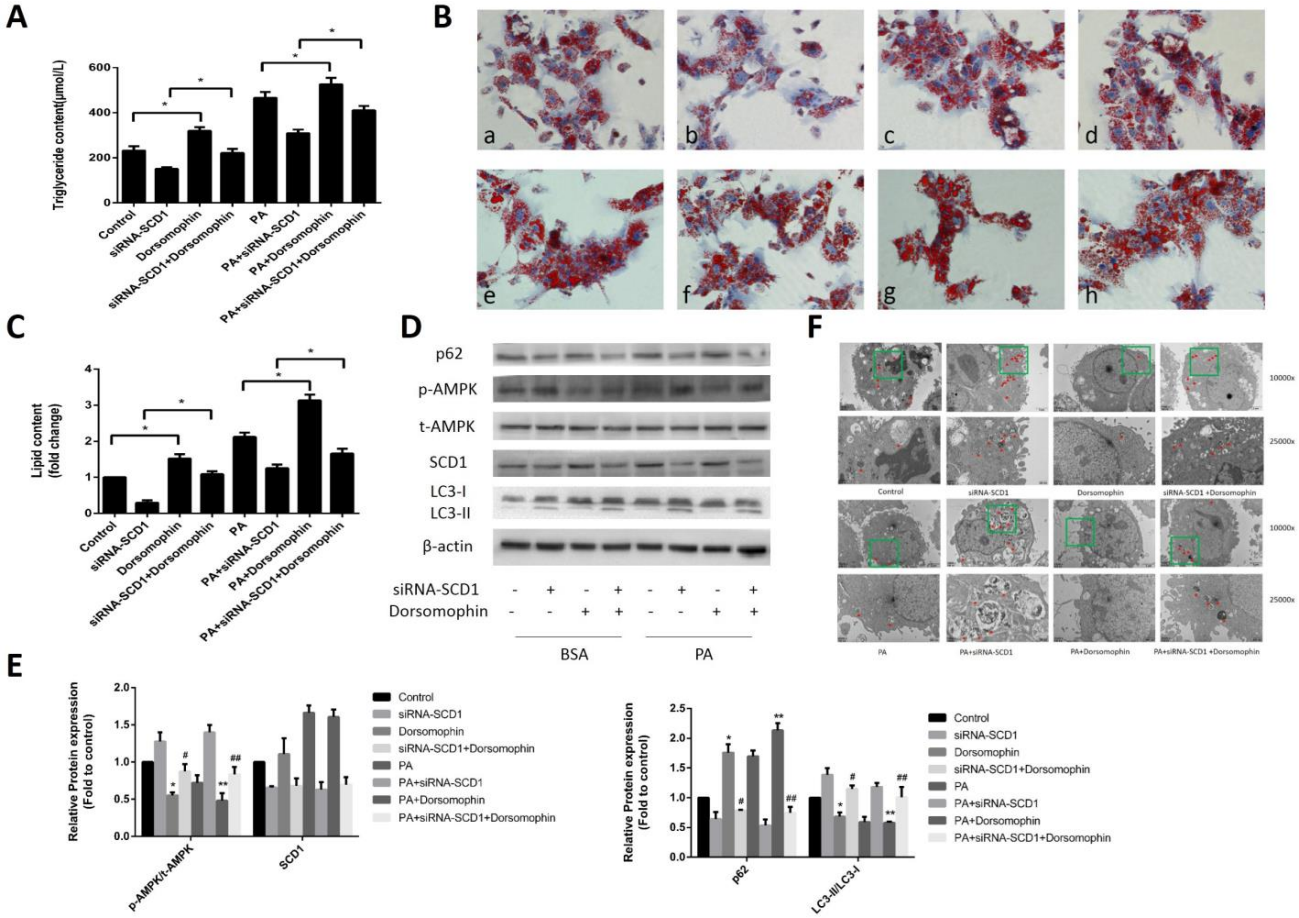
**Effects of cotreatment with siRNA-SCD1 and the AMPK inhibitor on lipid deposition and lipophagy in primary hepatocytes.** (**A**) TG levels were measured. (**B**) Primary hepatocytes were stained with Oil Red O. *a,* control group; *b,* siRNA-SCD1 group; *c,* Dorsomophin group; *d,* siRNA-SCD1+Dorsomophin group; *e,* PA group; *f,* PA+siRNA-SCD1 group; *g,* PA+Dorsomophin group; *h,* PA+siRNA-SCD1+Dorsomophin group. (**C**) The intracellular lipid content in each group was quantified. (**D**, **E**) Protein levels were determined by Western blotting. (**F**) Autophagosomes and autolysosomes in hepatocytes were observed by TEM. The red arrow indicates autophagosomes and autolysosomes. The data are presented as the means±SDs. *p < 0.05 versus control, #*p < 0.05* versus the siRNA-SCD1 group, **p < 0.05 versus the PA group, ##*p < 0.05* versus the PA+siRNA-SCD1 group.

Previous studies reported that inhibition of SCD1 expression leads to stimulation of AMPK signaling in various cancer cells [20–21]. In addition, as shown above, downregulation of SCD1 induced AMPK activation (Figure 2F, 2G) in primary hepatocytes. Because AMPK activation acts as a key positive regulator of autophagy, we investigated whether AMPK is involved in the activation of autophagy mediated by SCD1 inhibition in sodium palmitate-treated hepatocytes. We assessed changes in the lipid content in hepatocytes treated with siRNA-SCD1, Dorsomorphin (a selective AMPK inhibitor), and sodium palmitate as single agents or in combination. We observed that the intracellular TG levels (Figure 4A) and lipid droplet accumulation (Figure 4B, 4C) were increased in the PA+Dorsomorphin group compared with those in the PA group, and in the PA+siRNA-SCD1+Dorsomorphin group compared with those in the PA + siRNA-SCD1 group (*P* < 0.05). As shown in Figure 4D, 4E, treatment of hepatocytes with Dorsomorphin markedly decreased the phosphorylation of AMPK. The expression of SCD1 in the PA+Dorsomophin group did not change significantly compared with that in the PA group. The conversion of LC3-I to LC3-II was decreased in the PA+siRNA-SCD1+Dorsomorphin group compared with that in the PA+siRNA-SCD1 group but was increased compared to that in the PA+Dorsomorphin group. The opposite pattern was observed for the expression of p62. Furthermore, autophagosomes and other autophagic vacuoles in hepatocytes were directly observed by TEM. As shown in Figure 4F, hepatocytes treated with sodium palmitate contained fewer autophagosomes and autolysosomes than hepatocytes not treated with sodium palmitate. siRNA-SCD1 led to an increase in the number of autophagosomes and autolysosomes in hepatocytes. Inhibition of AMPK obviously decreased the number of autophagosomes and autolysosomes in hepatocytes treated with siRNA-SCD1. Collectively, these results strongly indicate that the induction of autophagy triggered by SCD1 inhibition in sodium palmitate-treated hepatocytes is related to the AMPK signaling pathway.

### Effects of cotreatment with SCD1-OE and the AMPK activator on lipid deposition and lipophagy in primary hepatocytes

**Figure 5:**
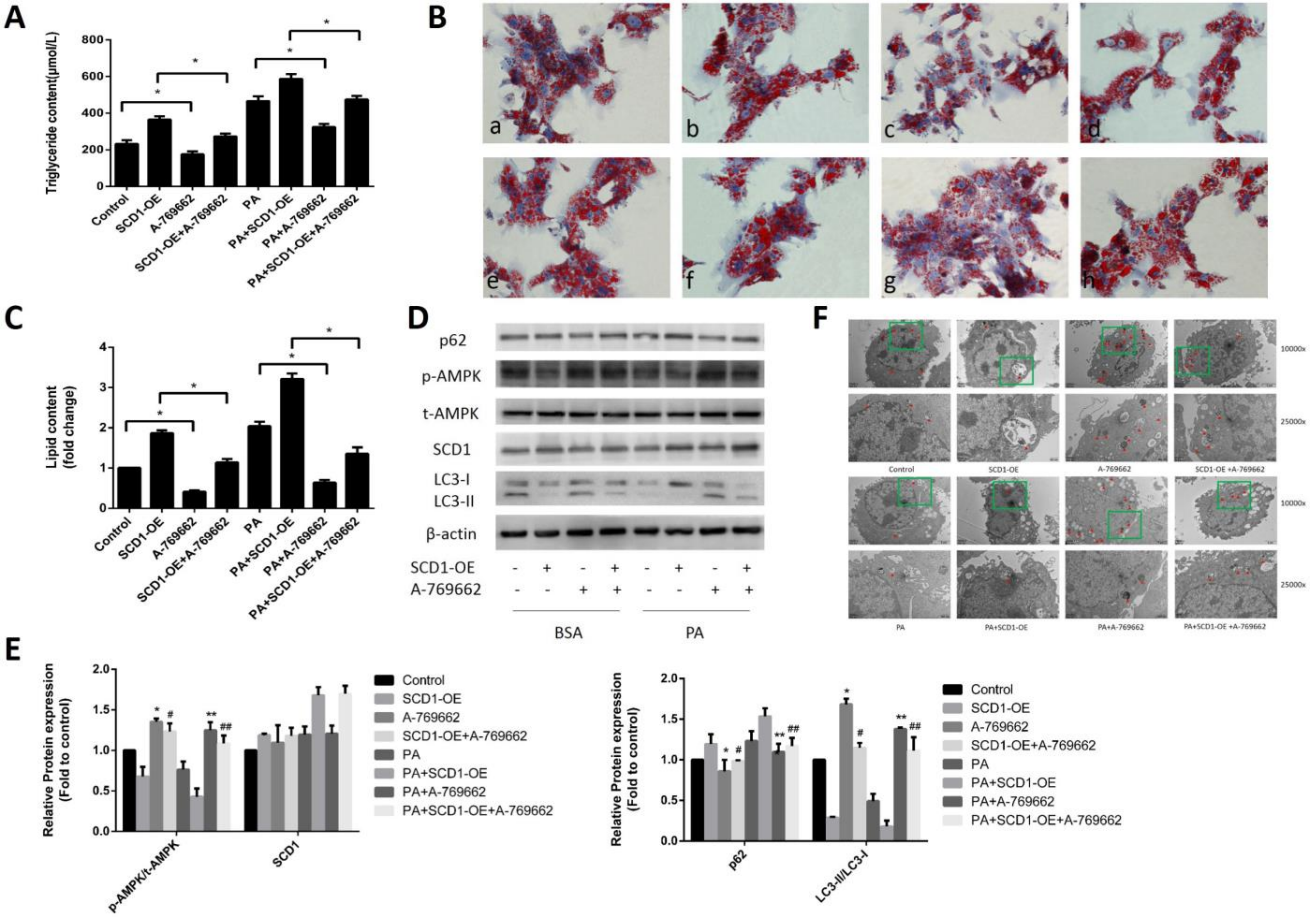
**Effects of cotreatment with SCD1-OE and the AMPK activator on lipid deposition and lipophagy in primary hepatocytes.** (**A**) TG levels were measured. (**B**) Primary hepatocytes were stained with Oil Red O. *a,* control group; *b*, SCD1-OE group; *c,* A-769662 group; *d,* SCD1-OE+A-769662 group; *e*, PA group; *f*, PA+SCD1-OE group; *g,* PA+A-769662 group; *h,* PA+SCD1-OE+A-769662 group. (**C**) The intracellular lipid content in each group was quantified. (**D**, **E**) Protein levels were determined by Western blotting. (**F**) Autophagosomes and autolysosomes in hepatocytes were observed by TEM. The red arrow indicates autophagosomes and autolysosomes. The data are presented as the means±SDs. *p < 0.05 versus control, #*p < 0.05* versus the SCD1-OE group, **p < 0.05 versus the PA group, ##*p < 0.05* versus the PA+SCD1-OE group.

To further determine whether the AMPK signaling pathway is responsible for the regulation of lipophagic activity by SCD1 in sodium palmitate-treated hepatocytes, we treated hepatocytes with SCD1-OE, A-769662 (a selective AMPK activator) and sodium palmitate as single agents or in combination. As shown in Figure 5A–5C, lipid droplet accumulation and intracellular TG levels were decreased in the PA+A-769662 group compared with those in the PA group and in the PA+SCD1-OE+A-769662 group compared with those in the PA+SCD1-OE group (*P* < 0.05). Treatment of hepatocytes with A-769662 increased the phosphorylation of AMPK (Figure 5D, 5E). The expression of SCD1 in the PA+A-769662 group did not change significantly compared with that in the PA group. The conversion of LC3-I to LC3-II was increased in the PA+SCD1-OE+A-769662 group compared with that in the PA+SCD1-OE group but was decreased compared to that in the PA+A-769662 group. The opposite pattern was observed for the expression of p62. We further measured the number of autophagosomes and other autophagic vacuoles in hepatocytes using TEM. As shown in Figure 5F, SCD1-OE led to a decrease in the number of autophagosomes and autolysosomes in hepatocytes. Conversely, activation of AMPK obviously increased the number of autophagosomes and autolysosomes in hepatocytes treated with SCD1-OE. Taken together, these data show that lipophagy was impaired by sodium palmitate via SCD1-mediated suppression of the AMPK signaling pathway in hepatocytes.

### Effects of in vivo inhibition of SCD1 expression on hepatic steatosis and activation of AMPK and lipophagy in mice

**Figure 6:**
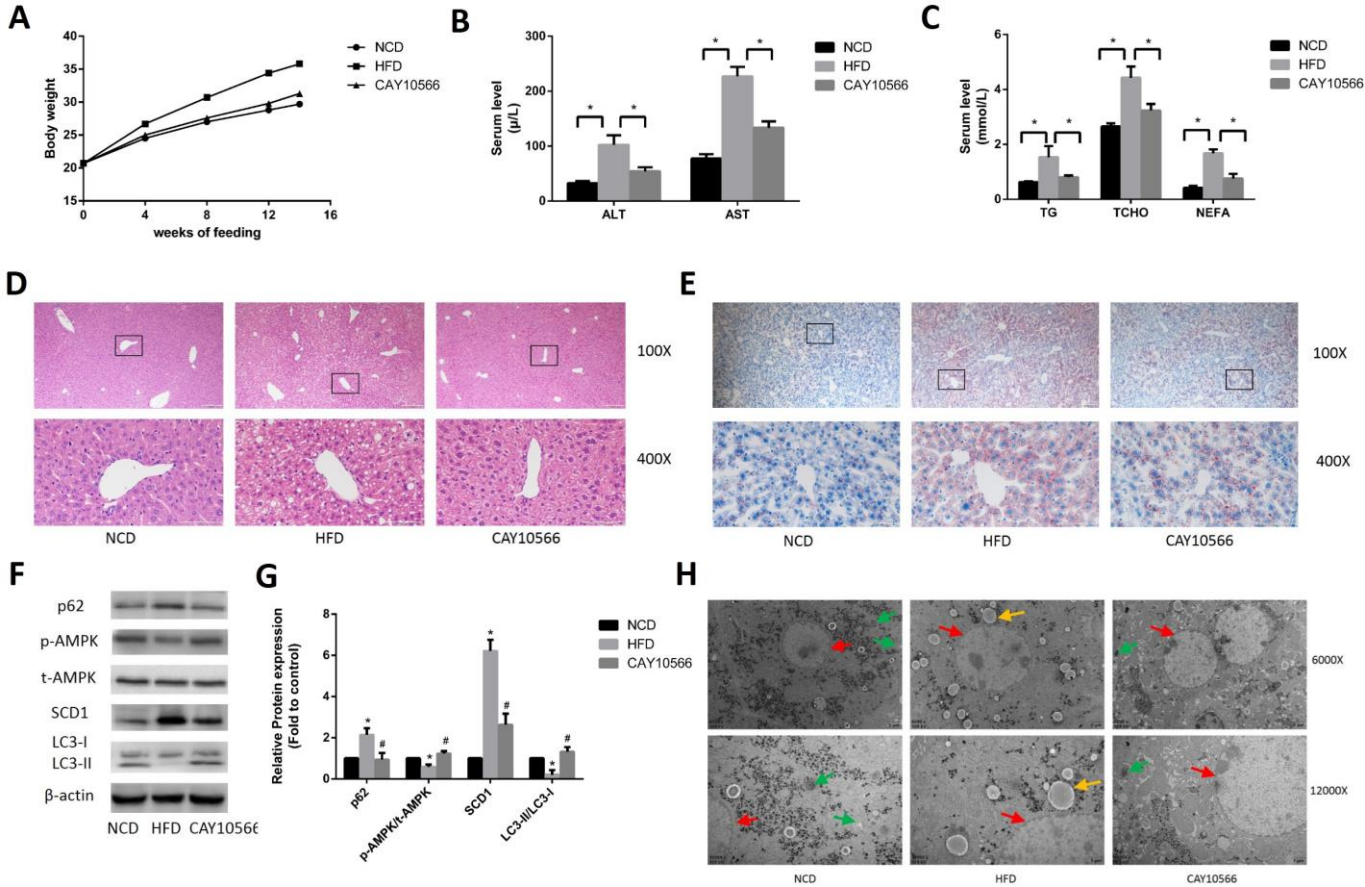
**Effects of in vivo inhibition of SCD1 expression on hepatic steatosis and activation of AMPK and lipophagy in mice.** (**A**) The body weights of mice in each group. (**B**) Levels of serum liver enzymes in mice after 14 weeks (n=6). (**C**) Levels of serum lipids in mice after 14 weeks (n=6). (**D**) H&E staining of mouse hepatic tissue (original magnification: 100X and 400X). (**E**) Oil Red O staining of mouse hepatic tissue (original magnification: 100X and 400X). (**F**, **G**) Protein levels were determined by Western blotting. (**H**) Autophagosomes and autolysosomes in hepatocytes in the liver tissues of mice were observed by TEM. The red arrows indicate hepatocytes, the yellow arrows indicate lipid droplets, and the green arrows indicate autophagosomes and autolysosomes. The data are presented as the means±SDs. *p < 0.05 versus the NCD group, #*p < 0.05* versus the HFD group.

To determine the effect of SCD1 on hepatic steatosis and activation of AMPK and lipophagy in a mouse model of NAFLD, C57BL/6 mice were fed a HFD for 14 weeks and treated or not treated with CAY10566, a specific SCD1 inhibitor. As shown in Figure 6A, the body weights of CAY10566-treated mice were significantly decreased compared to those of HFD-fed mice. In addition, the serum levels of ALT, AST, TG, TCHO and NEFA were significantly higher in the HFD group than in the NCD group, but significantly lower in the CAY10566 group than in the HFD group (Figure 6B, 6C). H&E staining showed that HFD-fed mice developed typical hepatocellular ballooning and inflammatory infiltration (Figure 6D). In addition, Oil Red O staining showed an abundance of lipid droplets in the livers of HFD-fed mice but not control mice (Figure 6E). However, compared to HFD-fed mice, CAY10566-treated mice exhibited significantly ameliorated hepatic steatosis and decreased hepatic lipid droplet accumulation (Figure 6D, 6E).

Western blot analysis (Figure 6F, 6G) demonstrated that SCD1 protein levels were significantly higher in the livers of HFD-fed mice than in the livers of NCD-fed mice, while CAY10566 treatment markedly decreased the SCD1 protein levels. The level of phosphorylated AMPK protein was decreased in the livers of HFD-fed mice, but was significantly increased by CAY10566 treatment. In addition, the conversion of LC3-I to LC3-II was decreased in the livers of HFD-fed mice but increased in the livers of CAY10566-treated mice compared with that in HFD-fed mice. In turn, p62 protein levels were increased in the livers of HFD-fed mice, but decreased in the livers of CAY10566-treated mice compared with those in HFD-fed mice. Furthermore, autophagosomes and autolysosomes in mouse hepatocytes were observed by TEM (Figure 6H). Of the three groups, the NCD group exhibited the highest number of autophagosomes and autolysosomes. No distribution of autophagosomes and autolysosomes was observed in the HFD group. The number of autophagosomes and autolysosomes in the CAY10566 group was decreased compared with that in the NCD group but significantly increased compared with that in the HFD group. Thus, these results suggest that SCD1 inhibition may protect mice from the effects of a HFD by inducing lipophagy through activation of the AMPK signaling pathway.

## DISCUSSION

**Figure 7:**
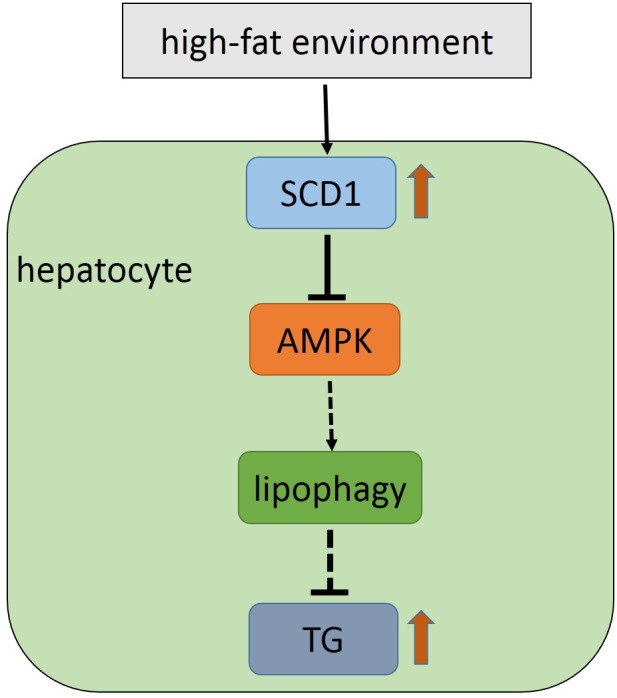
**Model for the SCD1-AMPK-lipophagy axis in hepatocyte in the high-fat environment.**

According to the classical theory of NAFLD pathogenesis, the multiple-hit theory, aberrant accumulation of TGs in the liver is the basis for the occurrence and development of NAFLD [22, 23]. In general, the causes of abnormal lipid deposition in hepatocytes can be condensed into the following three aspects: lipid entry into the liver is increased, de novo cellular lipid synthesis is enhanced, and lipid mobilization is reduced. SCD1 is a key rate-limiting enzyme in lipid metabolism and is involved in both lipid synthesis and mobilization. In addition, lipophagy plays a role in lipid mobilization and is a new approach to regulate lipid metabolism. AMPK is involved in the control of lipid metabolism by regulating the formation of autophagosomes during autophagy. However, our results revealed a correlation of above three genes in NAFLD, that the novel function of SCD1 in the regulation of lipophagy via AMPK signaling (Figure 7).

In our study, SCD1 expression was increased in both primary hepatocytes stimulated with sodium palmitate and in HFD-mice, while inhibition of SCD1 expression reduced lipid deposition and overexpression of SCD1 exacerbated lipid deposition. These results were consistent with those of previous studies and further confirmed that SCD1 is involved in the development of NAFLD [5, 6]. Previous studies on the role of SCD1 in lipid metabolism have focused on fatty acid synthesis and fatty acid oxidation. Recently, majority of researchers demonstrated that impaired autophagy had been linked to several metabolic liver disorders including NAFLD [24]. And the correlation between SCD1 and autophagy has attracted the attention of scholars. Ogasawara Y et al first confirmed that SCD1 is required for the early stages of autophagosome formation [25]. However, whether SCD1 can regulate lipid metabolism through lipophagy to participate in the development of NAFLD has not been reported.

In addition, Tan et al. found inhibition of SCD1 activity by either a chemical inhibitor or genetic knockdown resulted in an increase of autophagic flux only in the tsc2 −/− MeFs [26]. In our previous studies, we demonstrated that SCD1 can negatively regulate autophagy in HCC cells [19]. Similarly, this study confirmed that lipophagy was suppressed and SCD1 expression was increased simultaneously in the NAFLD models of both cells and mice. When SCD1 expression was inhibited and overexpressed respectively, we observed the enhanced lipophagy and suppressed lipophagy respectively. Our results indicate that SCD1 expression is negatively correlated with lipophagy in fatty liver. However, there were still some questions about how SCD1 regulate lipophagy.

Previous studies suggested that SCD1 has a regulatory effect on the AMPK signaling pathway. In hepatocytes with excessive lipid deposition, specific knockout of SCD1 or inhibition of SCD1 expression, the AMPK signaling pathway can be activated, leading to a decrease in lipid content and ultimately reducing the degree of hepatic steatosis [17, 19]. The autophagy machinery involves several multi-protein complexes that control every step, and AMPK regulates various aspects of this machinery [27]. In HCT-116 cells, the SCD1 inhibitor T-3764518 induces AMPK phosphorylation, which activates the AMPK pathway, resulting in reduced fatty acid synthesis and enhanced autophagy [28]. In addition, previous studies by our research group found that the inhibition of SCD1 leads to autophagy-induced apoptosis via AMPK signaling in human HCC cells [19]. Our present study also showed that AMPK expression decreased accompanied with the increase of SCD1 expression and the decreased of lipophagy in fatty liver models. Meanwhile, we also found that both the AMPK expression and the lipophagy had the same change as SCD1 expression was inhibited or overexpressed. To further explore the mechanism by which SCD1 regulates lipophagy, we simultaneously altered the expression levels of SCD1 and AMPK and found that AMPK inhibitors and agonists did not significantly affect SCD1 expression. After downregulation of SCD1 expression, the addition of AMPK inhibitors attenuated lipophagy and exacerbated lipid deposition. After upregulation of SCD1 expression, the addition of AMPK agonists enhanced lipophagy and reduced lipid deposition. To further validate the role of SCD1 in vivo, we treated HFD-fed mice with SCD1 inhibitors and found that inhibition of SCD1 expression in vivo activates AMPK, enhances lipophagy, and reduces abnormal lipid deposition in the liver. The results of this study confirmed that SCD1 can regulate lipophagy through the AMPK signaling pathway to affect lipid metabolism, suggesting that SCD1 is the entry point for breaking the “vicious circle” of lipophagy and lipid metabolism.

Collectively, the results of this study provide the first demonstration that inhibition of SCD1 ameliorates hepatic steatosis by inducing AMPK-mediated lipophagy, suggesting the novel function of SCD1 in hepatic lipogenesis and that the SCD1-AMPK-lipophagy pathway may be a potential therapeutic target for NAFLD. However, there are also some limitations in this study. NAFLD has a profound sexual dimorphism [29], we should repeat our experiments further by taking into account this specific feature of NAFLD. And in the following experiment, we need to further explore how SCD1 regulates the AMPK-lipophagy pathway.

## MATERIALS AND METHODS

### Isolation and treatment of primary hepatocytes

Isolation of primary hepatocytes was performed by collagenase digestion and by the two-step Percoll gradient method with slight modifications as described previously [30]. Briefly, primary hepatocytes were isolated from a 6- to 8-week-old male C57BL/6 mouse. The liver was perfused with prewarmed (37 °C) Dulbecco’s Hank’s balanced salt solution (pH 7.6, HyClone, USA) and collagenase type IV (0.5 mg/mL, Sigma, USA). The digested liver was then dissected and the isolated cells were washed with precooled (4 °C) Hank’s balanced salt solution (HyClone, USA). The cell pellet was then added to DMEM containing 10% fetal bovine serum at a density of 1.0 X 106 cells/mL and cultured at 37 °C in a humidified incubator containing 5% CO2. Twenty-four hours later, nonadherent cells were removed, and fresh medium was added to adherent cells. Primary hepatocytes were treated with 324 μM sodium palmitate (PA) for 24 h. Sodium palmitate was prepared with bovine serum albumin (BSA) solution as previously described [31]. Subsequently, according to experimental requirements, the cells were treated with 10 mM Dorsomorphin (an AMPK inhibitor) or A-769662 (an AMPK activator) for 2 h.

### Transfection assay

siRNA-SCD1 was purchased from GenePharma Biotechnology Co. (China). SCD1 overexpression adenoviruse (SCD1-OE) adenoviruse was generated by Abmgoodchina Inc. (China). siRNA was transfected with siRNA Transfection Reagent GM siRNA-mate (GenePharma Biotechnology Co., China) into primary hepatocytes for 48 h, and the transfection efficiency was greater than 80%. Adenoviral infection was performed according to the manufacturer’s instructions, and the infection efficiency was greater than 80% after 48 h of infection. If necessary, after 48 h of transfection, cells were treated with sodium palmitate, Dorsomorphin or A-769662 for the indicated time and harvested for subsequent processing.

### Animal experiments

C57BL/6 mice (6 weeks old, male, n=30) were obtained from the Animal Research Center of Chongqing Medical University. All mice were housed on a 12:12 h light: dark cycle at 25°C and had free access to food and water. Mice were fed with standard laboratory chow diet (NCD) (n= 10) or a HFD (n=20) (Research Diets, D12492) for 14 weeks. Half of the HFD-mice (n=10) received an intraperitoneal injection of 7.5 mg/kg CAY10566 (SCD1 inhibitor) once every three days for 14 weeks. CAY10566 was purchased from Cayman Chemical. All mice were sacrificed after 14 weeks of feeding. Body weights were measured weekly. Blood samples were collected for biochemical assays. Liver tissue samples were collected for Oil Red O staining, hematoxylin and eosin (H&E) staining, transmission electron microscopy (TEM) and Western blot analysis. In addition to the special requirements, the extracted tissues were immediately frozen in liquid nitrogen and stored at -80 °C until used for analysis. All animal studies were conducted in accordance with guidelines approved by the Institutional Animal Care and Use Committee of Chongqing Medical University.

### Biochemical assays

Blood samples were collected by retroorbital bleeding and serum was centrifuged at 2000 rpm for 10 min. The levels of serum alanine aminotransferase (ALT), aspartate aminotransferase (AST), nonesterified fatty acids (NEFA), total cholesterol (TCHO) and TGs were determined using an automatic biochemical analyzer (Beckman, the USA).

### Histological examination

Liver tissues were fixed using 4% paraformaldehyde, dehydrated in an alcohol gradient and subsequently transferred to dimethylbenzene. Tissues were embedded in paraffin and cut into 3- to 5-μm-thick sections. Sections were deparaffinized in xylene and rehydrated in a series of decreasing concentrations of ethanol. Sections were then stained with H&E for histological analysis. Stained sections were observed with an optical microscope (Nikon, Japan).

### Measurement of intracellular TGs

After treatment of primary hepatocytes, TGs were extracted using RIPA buffer and measured by a commercial kit (Applygen Technologies Inc., China) according to the manufacturer’s instructions. Briefly, the lysate suspension was incubated at 70 °C for 10 min and centrifuged at 2000 rpm for 5 min. Then, the TG level in supernatant was measured by an enzymatic method.

### Oil Red O staining

After treatment, primary hepatocytes and frozen sections of liver were stained with Oil Red O for evaluation of lipid content. Samples were fixed with 4% paraformaldehyde for 30 min, followed by washing with 60% isopropanol for 2 min. Samples were then stained with freshly diluted Oil Red O solution for 5- to 10- min at room temperature. Cell nuclei were counterstained with hematoxylin after washing with 60% isopropanol. Oil Red O-stained lipid droplets were observed by bright-field microscopy. Images of Oil Red O staining were analyzed using Image-Pro® Plus 6.0 (Media Cybernetics Corp., Rockville, Maryland, USA). Three representative fields were randomly selected from each group for calculation of the integrated optical density (IOD) value of the lipid droplets, and the intracellular lipid content was compared between groups.

### TEM

Primary hepatocytes (>106) and liver sections (<1 mm3) were collected and fixed in 2.5% glutaraldehyde in cacodylate buffer. Samples were then postfixed in 1% osmium tetroxide, dehydrated and infiltrated with an increasing concentration gradient of ethanol and propylene oxide. Then, samples were embedded and solidified, cut into 50-60--nm slices, and stained with 3% uranyl acetate and lead citrate. Slices were then observed via TEM.

### Western blotting analysis

Total protein was extracted with lysis buffer (KeyGen BioTech, China) supplemented with 1 mM phenylmethanesulfonyl fluoride (PMSF) according to the manufacturer’s instructions. The protein concentration was determined using a bicinchoninic acid (BCA) protein assay kit (KeyGen BioTech, China). Forty micrograms of protein per sample was separated by 8-15% sodium dodecyl sulfate-polyacrylamide gel electrophoresis (SDS-PAGE) and transferred to polyvinylidene difluoride (PVDF) membranes (Millipore, Massachusetts, USA). After blocking in skim milk for 1 h, membranes were incubated with primary antibodies overnight at 4 °C, followed by incubation with horseradish perox-idase (HRP)-conjugated secondary antibody. The following primary antibodies were used: anti-AMPK (1:1000; CST), anti-phospho-AMPK (1:1000; CST), anti-SCD1 (1:1000; Abcam), anti-p62 (1:1000; Sigma), anti-LC3 (1:1000; Sigma) and anti-β-actin (1:1000; Sigma). Protein bands were then visualized using enhanced chemiluminescence reagents (Millipore, USA). Fold density quantitation was done using Quantity One software (Bio-Rad Laboratories, Hercules, CA).

### Statistical analysis

Experimental results were analyzed with SPSS 17.0 software. All quantitative data are expressed as the means ± SDs and were considered statistically significant at a probability (p)-value of < 0.05. Differences between two groups were compared using Student’s t test. Multiple group comparisons were assessed using one-way analysis of variance (ANOVA) followed by the Student-Newman-Keuls test.
